# Effect of tetracyclines on pulpal and periodontal healing after tooth replantation: a systematic review of human and animal studies

**DOI:** 10.1186/s12903-021-01615-y

**Published:** 2021-06-05

**Authors:** Mingmei Meng, Yandi Chen, Huidi Ren, Qiong Zhang, Song Chen, Xuedong Zhou, Jing Zou

**Affiliations:** 1 State Key Laboratory of Oral Diseases and National Clinical Research Center for Oral Diseases, West China Hospital of Stomatology, Sichuan University, Sichuan, China; 2 Departments of Pediatric Dentistry and Orthodontics, West China Hospital of Stomatology, Sichuan University, Sichuan, China; 3 Department of Endodontics, West China Hospital of Stomatology, Sichuan University, Sichuan, China

**Keywords:** Tetracyclines, Tooth replantation, Pulpal healing, Periodontal healing

## Abstract

**Background:**

Pulpal and periodontal healing are two main concerns of delayed replantation of avulsed teeth. The objective of this review was to evaluate the effectiveness of topical and systemic application of tetracyclines on pulpal and periodontal healing after tooth replantation.

**Methods:**

A comprehensive electronic search was conducted in six databases. This systematic review was carried out according to Cochrane Handbook and the Preferred Reporting Items for Systematic Reviews and Meta-Analyses (PRISMA) statement.

**Results:**

After exclusion of 246 irrelevant papers, 14 animal studies and one human study were included in this review. The human study showed that avulsed permanent teeth treated with doxycycline did not show a better clinical outcome for pulp and periodontal healing compared with treatment with normal saline. As for animal studies, significant more pulpal healing was observed in immature teeth treated with topical doxycycline in two researches, while another one study showed that there is no difference between teeth treated with normal saline and teeth treated with doxycycline. Systemic doxycycline exerted no significant effect on pulpal revascularization illustrated by one research. Only one out of four articles illustrated the positive effect of systemic tetracyclines on periodontal healing. One paper reported that intracanal application of demeclocycline promoted favorable periodontal healing. Two articles showed topical doxycycline contributed to favorable periodontal healing, while five studies showed no significant effect of topical tetracyclines on periodontal healing.

**Conclusions:**

As a result of data heterogeneity and limitations of the studies, the effect of topical or systemic application of tetracyclines on pulpal and periodontal healing is inconclusive. More studies are required to get more clinically significant conclusions.

## Background

Dental trauma, occurring frequently in patients between 7 and 15 years of age, affects the maxillary central incisors most [1]. Avulsion, the complete luxation of a tooth from its alveolar fossa, may cause a variety of complications related to pulp and periodontal tissues. The development of pulp necrosis, pulp canal obliteration, arrested or incomplete root formation, external resorption, inflammatory resorption, permanent replacement resorption, transient replacement resorption, internal root resorption, loss of marginal attachment and tooth loss are possible healing complications secondary to tooth avulsion [2, 3].

Factors related to pulpal healing, root growth and periodontal healing after tooth avulsion have been widely studied. Stage of root development, the distance from the apical foramen to the pulp horns, the time and condition of extra-alveolar storage, and atopic feature are issues frequently considered [4–11]. It is widely accepted that suitable management for first-aid measures and immediate replantation of avulsed tooth is the best for prognosis. Delayed replantation and unphysiological storage is followed by low survival [12].

Attempts have been made to facilitate greater pulp and periodontal healing, and greater lifetime of the replanted teeth. Thymosin alpha 1, enamel matrix derivative, fibroblast growth factor, triamcinolone, bisphosphonates dexamethasone, 3Mix (the mixture of ciprofloxacin, metronidazole, and minocycline), Cathepsin K inhibitor and fluoride are medicines tried in different models to provide short-term and long-term benefits in the replantation of avulsed teeth [13–22]. They might exert their beneficial effect on replanted teeth by inhibiting the activity of osteoclasts, controlling the inflammation through decreased C-reactive protein, or favoring the formation of new periodontal ligament.

Minocycline, one component of 3Mix, has been reported to suppress osteoclast differentiation and accelerate odontoblast like cell differentiation in intentionally delayed tooth replantation in mice, improving pulpal healing after tooth injuries [20]. Other tetracyclines have also been applied in avulsed teeth topically or systemically in the purpose of promoting pulp revascularization and periodontal healing [21, 23–37]. Tetracyclines, a group of broad-spectrum antibiotics, either natural or semi-synthetic, exert their antimicrobial activity by inhibiting protein synthesis. They have been widely used in stomatology, such as regenerative endodontics, white spongy naevus, periodontitis, peri-implantitis, herpes labialis, recurrent aphthous stomatitis [38–43]. The mechanism of action in these conditions is not totally understood, but its efficacious antibiotic properties, the inhibitory effect of collagenase and osteoclastic activity, and its enhancement of fibroblastic attachment to facilitate periodontal regeneration might contribute to these clinical effects [44–48].

An animal study suggested no beneficial effect of systemic use of doxycycline in facilitating complete pulp revascularization and inhibiting micro-organisms in the pulp lumen [23]. Another research had different results, revealing that the topical application of doxycycline could increase the frequency of complete pulp revascularization and decrease the frequency of micro-organisms in the pulpal lumen, ankylosis and inflammatory root resorption [24]. Therefore, a systematic review of available knowledge is in need to comprehensively evaluate the effect of tetracyclines on pulp revascularization and periodontal healing in replanted teeth, with the intent of getting instructive information for clinical practice.

## Material and methods

This systematic review was conducted in accordance with Cochrane Handbook for Systematic Review of Interventions and Preferred Reporting Items for Systematic Reviews and Meta-Analyses (PRISMA). Two reviewers (MM and YC) conducted the search and the work of extracting data, and assessed the risk of bias and eligibilities of the retrieved articles independently and in duplicate. Any disagreement was resolved by discussing with a third reviewer (JZ).

### Search strategy

An extensive electronic search was made through PubMed, Embase (via Ovid), Web of Science, Cochrane Central Register of Controlled Trials (CENTRAL), and ProQuest research Library (PRL). The electronic search was conducted in different databases from their date of inception up to April 2021, with no language limitation. The following search terms were used in Pubmed: (“tetracyclines” [MeSH Terms] OR “tetracyclines” [All Fields] OR “chlortetracycline” [All Fields] OR “demeclocycline” [All Fields] OR “doxycycline” [All Fields] OR “lymecycline” [All Fields] OR “methacycline” [All Fields] OR “minocycline” [All Fields] OR “oxytetracycline” [All Fields] OR “rolitetracycline” [All Fields] OR “tetracycline” [All Fields] OR “tigecycline” [All Fields]) AND (“tooth replantation” [MeSH Terms] OR “tooth replantation” [All Fields]). The reference lists of the relevant studies were also searched to identify any additional relevant articles.

### Study inclusion and exclusion criteria

Inclusion criteria were as follows:

1. Types of studies. Studies that estimated the efficacy of tetracyclines on pulp revascularization and periodontal healing after tooth replantation were included. For human studies, both randomized and non-randomized clinical trials were included. For animal studies, the experimental group should undergo the same procedure as the control group, while the additional application of tetracyclines was given in the intervention group.
2. Types of subjects. Subjects included in this review should be animals or human beings. For the former, experimenters should be approved by the appropriate Ethics Committee for Animal Experiments. For the latter, the human subjects signed an informed consent before the research procedures.
3. Types of interventions. Interventions should be application of tetracyclines to the avulsed teeth. Compounded trials, in which replanted teeth in the experimental group were subjected to some other medications besides tetracyclines or some chemical compounds including other ingredients, were excluded.

Exclusion criteria were as follows:

Reviews, case reports, cohort studies, retrospective studies, descriptive studies, letters, opinion articles, and abstracts were excluded.

### Study inclusion

Two reviewers (MM and YC) independently conducted the search, screening the titles and abstracts. The full texts were further evaluated for studies appearing to meet the inclusion criteria or when information given by the abstracts was insufficient to judge whether the articles met the inclusion criteria or not. This procedure was also conducted independently and in duplicate by two review authors (MM and YC). A third person (JZ) from our team provided input as needed.

### Assessment of risk of bias

Risk of bias assessment was undertaken according to Cochrane Handbook for Systematic Reviews of Interventions. The features of interest in the standard ‘‘Risk of bias’’ table of Cochrane review were as follows: random sequence generation (selection bias), allocation sequence concealment (selection bias), blinding of participants and personnel (performance bias), blinding of outcome assessment (detection bias), incomplete outcome data (attrition bias), selective outcome (reporting bias), and other potential sources of bias. Each entry of these features addressed a specific feature of the included studies. They were judged as ‘‘low risk’’, ‘‘high risk’’, or “unclear risk’’, with the last category indicating either lack of information or uncertainty over the potential for bias. Two independent reviewers (MM and YC) conducted the assessment of risk of bias in duplicate. Disagreement was resolved by discussing with a third reviewer (JZ).

### Data extraction

Basic information of the included studies were extracted and recorded independently and in duplicate by two reviewers using a data extraction form, including participants details, teeth replanted, stage of root development, the status of root canal during the experiment (endodontically treated or not), extra-alveolar time, storage conditions, splinting or not, application of tetracyclines, duration of study, observation methods, observation content, interventions for each group and the final conclusions.

## Results

### Search results

**Fig. 1:**
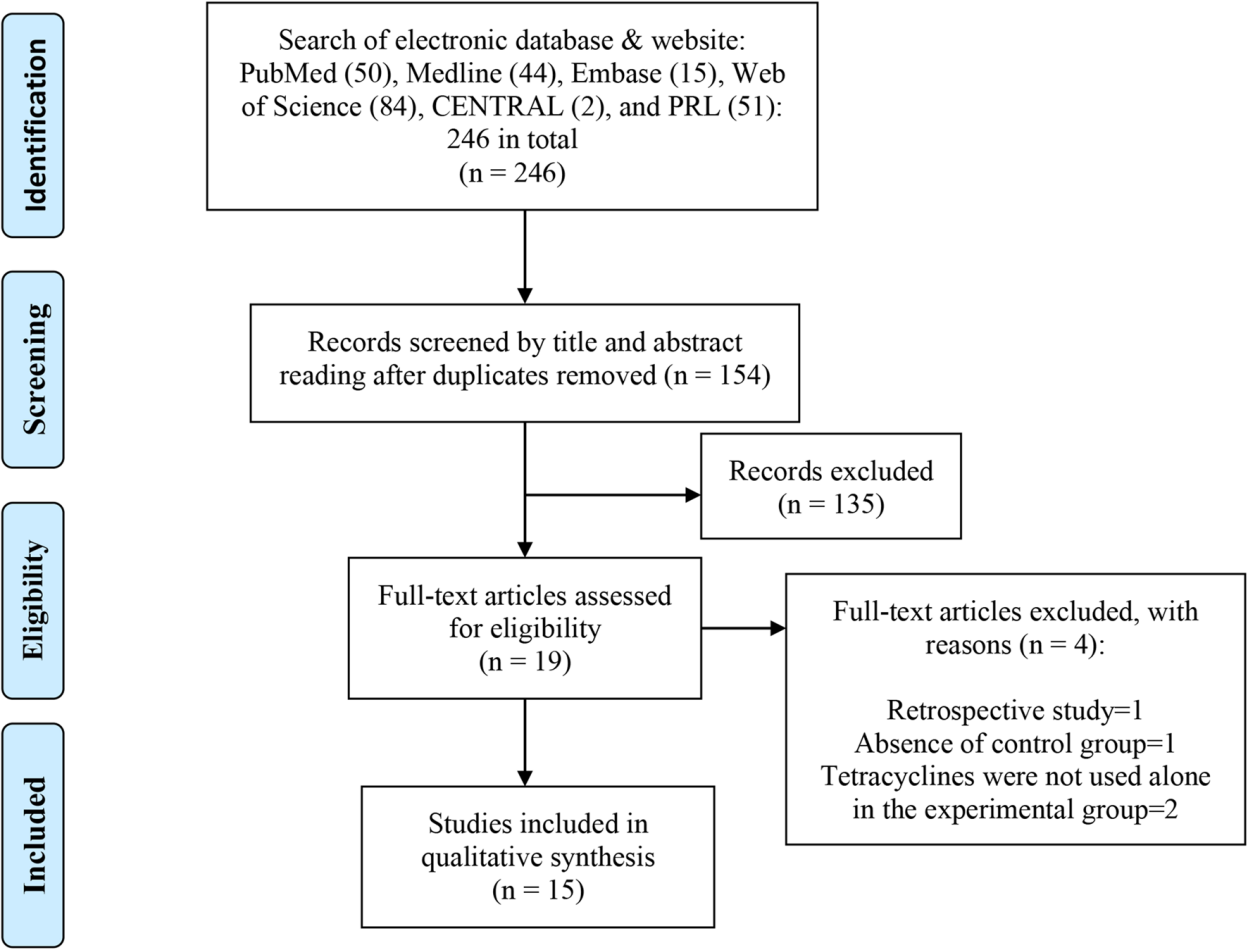
Systematic search and selection strategy (flow chart)

The primary search resulted in 246 articles. 227 studies did not meet the inclusion criteria and were excluded after reading of titles and abstracts. Four papers were excluded after evaluation of the full texts [30, 31, 37, 49]. There were 15 articles brought into this systematic review after applying the exclusion and inclusion criteria. The procedures of electronic searching are presented in Fig. 1.

### Characteristics of included studies

All articles that met our inclusion criteria were animal studies. There were ten studies evaluating the effect of topical application of tetracyclines on pulp and periodontal healing [21, 24, 27–29, 34–36, 50, 51]. Five studies focused on the influence of systemic tetracyclines after tooth replantation [23, 25, 26, 32, 33]. The duration of these studies ranged from 7 days to 6 months. Most teeth were kept dry. And teeth were kept wet intraorally in saliva in two papers [23, 24]. The extra-alveolar time ranged from less than 5–60 min. More information about the characteristics of the included studies is illustrated in Table 1.

**Table 1:** General characteristics of selected studies

Study ID	Subjects (n)	Teeth replanted (n) and the stage of root development	The status of root canal	Extra-alveolar time and storage conditions	Splinting or not	Duration of study
Cvek et al. [23]	Monkeys (n = 32)	Immature, maxillary incisors (105 teeth)	Untreated	30 or 60 min, dry or wet	Splinting with a band of a composite material	6–8 weeks
Sae-Lim et al. [25]	Beagles (n = 5)	Mature, lateral incisors, first and third premolars (30 roots)	Root canals, infected by dental plaque	less than 5 min	Splinting with resorbable sling sutures	5–6 months
Sae-Lim et al. [26]	Beagles (n = 4)	Mature, lateral incisors, the first and third premolars (31 roots)	Endodontic treatment	60 min, dry	Splinting with resorbable sling sutures	12 weeks
Cvek et al. [24]	Monkeys (n = 47)	Immature, maxillary incisors (171 teeth)	Untreated	30 or 60 min, dry or wet	Splinting whit a band of a composite material	6–8 weeks
Yanpiset et al. [29]	Mongrel dogs (n = 4)	Immature, 48 incisors and 48 premolars	Untreated	5 min, dry	No fixation	3 months
Ma et al. [27]	Monkeys (n = 7)	Mature, incisors and mandibular posterior teeth (32 roots)	Endodontically treated	60 min, dry	No fixation	12 weeks
Ritter et al. [28]	Mongrel dogs (n = 3)	Immature, 22 incisors and 12 premolars	Untreated	5 min, dry	No fixation	60 days
Bryson et al. [34]	Mongrel dogs (n = 4)	Mature, premolar roots (69 roots)	Endodontically treated	60 min, dry	No splinting	4 months
Chen et al. [21]	Beagles (n = 3)	Mature, premolar roots (24 roots)	Endodontically treated	60 min, dry	No splinting	4 months
Gomes et al. [32]	Wistar Rats (= 60)	Maxillary right incisor (n = 60)	Endodontically treated	60 min, dry	No splinting	7, 15, and 30 days after replantation
Melo et al. [33]	Wistar Rats (n = 60)	Maxillary right incisor (n = 60)	Untreated	5 min, immersed in saline	No splinting	7, 15, and 30 days after replantation
Bjorvatn et al. [36]	Beagles (n = 5)	Mature, mandibular incisors (n = 9)	Untreated	45 min, dry	Non-rigid splinting with dental floss	4 weeks after replantation
Selvig et al. [35]	Beagles (n = 5)	Mature, mandibular incisors( n = 10)	The middle part of the root was then root-planed extensively, leaving the cervical and apical root surface areas uninstrumented	45 min, dry	No fixation	21 days after replantation
Liu et al. [50]	Human (n = 38)	Mature and immature teeth, (n = 44)	Immature teeth were replanted without endodontic treatment, and endodontic treatment was indicated for those mature teeth with extra-oral time longer than 2 h before replantation	Extra-alveolar time and storage conditions varied in different cases	Splinting with composite resin	About 28 months after replantation
Nam et al. [51]	Sprague–Dawley rats (n = 20)	maxillary first molars with complete root formation (n = 40)	Untreated	dry for 5 min or 60 min in Hank's balanced salt solution (HBSS)	No splinting	8 weeks after replantation

### Methodological and quality assessment

Randomization was performed in three studies [23, 28, 29]. Blinding of outcome assessment was carried out in two studies [21, 34]. Five articles received a moderate risk of bias [21, 23, 28, 29, 34]. Ten articles showed a high risk of bias [24–27, 32, 33, 35, 36, 50, 51]. Risk of bias assessment according to the Cochrane Handbook for Systematic Reviews of Interventions for each study is presented in Table 2.

**Table 2:** Quality assessment of the included studies

Studies	Random sequence generation	Allocation concealment	Blinding of participants and personnel	Blinding of outcome assessment	Incomplete outcome data	Selective reporting	Other bias
Cvek et al. [23]	+	?	+	_	+	+	+
Sae-Lim et al. [25]	_	?	+	_	+	+	+
Sae-Lim et al. [26]	_	?	+	_	+	+	+
Cvek et al. [24]	_	?	+	_	+	+	+
Yanpiset et al. [29]	+	?	+	_	+	+	+
Ma et al. [27]	_	?	+	_	+	+	+
Ritter et al. [28]	+	?	+	_	+	+	+
Bryson et al. [34]	_	?	+	+	+	+	+
Chen et al. [21]	_	?	+	+	+	+	+
Gomes et al. [32]	_	?	+	_	+	+	+
Melo et al. [33]	_	?	+	_	+	+	+
Bjorvatn et al. [36]	_	?	+	_	+	+	+
Selvig et al. [35]	_	?	+	_	+	+	+
Liu et al. [50]	_	_	_	_	+	+	+
Nam et al. [51]	_	?	+	_	+	+	+

+ , low risk of bias; _, high risk of bias; ?, unclear risk of bias

### Effect of tetracyclines on pulpal and periodontal healing

The basic procedure conducted in the control group was similar with the experimental group. Only the additional application of tetracyclines was given in the intervention group. And the specific operation for the intervention and control group in different studies was summarized in Table 3. The administration and dosage of tetracyclines, methods used to evaluate the effect of tetracyclines on pupal and periodontal healing, and the outcomes of each study was also shown in Table 3.

**Table 3:** Intervention and outcomes of included studies

Study ID	Intervention and control group	Administration and dosage of tetracyclines	Observation	Outcomes
Cvek et al. [23]	C: extraction; extra-alveolar storage; replantation; E: additional systemic application of doxycycline	Doxycycline, 4 mg/kg intravenously at the time of anesthesia, approximately 20 min before extraction and, 2 mg/kg intramuscularly for 5 consecutive days after replantation	Hematoxylin–eosin stain, modified Gram stain, modified Mallory stain; the presence and amount of vital tissue, occurrence of inflammatory changes and presence of bacteria in the pulpal lumen	The prophylactic, systemic treatment with doxycycline had no effect on the frequency of pulp revascularization nor on the occurrence of inflammatory changes
Sae-Lim et al. [25]	C: pulp infection; hemisected; extraction; 5 mm fissure of the buccal or lingual mid-roots were shaved; replantation E: additional systemic application of tetracycline hydrochloride	Tetracycline hydrochloride, 20 mg/kg orally, on the day of extraction/replantation and for the following 6 days, three times a day	Hematoxylin–eosin stain; the appearance of the root surface was evaluated and classified as healed or showing the presence of inflammatory root resorption	Significantly more complete healing and less inflammatory root resorption in tetracycline group than the control group
Sae-Lim et al. [26]	C: extraction; extra-alveolar storage; replantation; E: additional systemic application of tetracycline hydrochloride	Tetracycline hydrochloride, 20 mg/kg, orally, immediately after replantation and for the following 6 days, three times a day	Hematoxylin–eosin stain; the appearance of the root surface was evaluated and classified as healed or showing the presence of replacement or inflammatory root resorption	Complete healing for the tetracycline was much higher than the control group, but it was not statistically different
Cvek et al. [24]	C: extraction; extra-alveolar storage; replantation; E: immersion in doxycycline for 5 min before replantation	1 mg doxycycline in 20 ml sterile physiologic saline	Hematoxylin–eosin stain, modified Gram stain; the presence and amount of vital tissue, occurrence of inflammatory changes and presence of bacteria in the pulpal lumen	Increased frequency of complete pulp revascularization and decreased frequency of micro-organisms in the pulpal lumen, ankylosis and inflammatory root resorption was observed in the doxycycline group
Yanpiset et al. [29]	C: extraction; extra-alveolar storage; replantation; E: immersion in doxycycline for 5 min before replantation	1 mg doxycycline in 20 ml saline	Radiographic evaluation and hematoxylin–eosin stain; assessment of the presence/absence of vital pulp tissue above the cervical margin of the tooth	Soaking for 5 min in doxycycline significantly increased the revascularization rate
Ma et al. [27]	C: endodontically treated; extraction; extra-alveolar storage; replantation; E: rinse with 5 ml saline and soaked in 1 ml 50 mg/ml minocycline for 5 min before replantation	Minocycline, 50 mg/ml	Hematoxylin–eosin stain; evaluation of the periodontal healing as healing, inflammatory resorption, or replacement resorption	Topical application of minocycline increased occurrence of complete healing slightly, but the difference was not statistically signifiant
Ritter et al. [28]	C: extraction; extra-alveolar storage; immersion in saline for 5 min; replantation; E: covered with minocycline hydrochloride microspheres for 5 min or soaked in doxycycline solution for 5 min before replantation	Covered with minocycline hydrochloride microsphere or soaked in doxycycline solution	Post-replantation radiographs 6 days after replantation, laser Doppler 7, 15, 25, 35, 45, 60 days after replantation, and H&E evaluation after sacrifice; Pulp revascularization	Minocycline-treated specimens presented a significantly higher number of vital teeth than saline-treated specimens. The number of vital teeth in doxycycline-treated specimens was higher than saline-treated specimens, but no statistical significance was observed
Bryson et al. [34]	C: instrumented; filled with gutta percha; hemisected; extracted; dried; replanted without minocycline E: coated with minocycline and then replanted	Coated with minocycline, no mention about the concentration	Hematoxylin–eosin stain; extent of root resorption and type of interface between the root and surrounding tissue (Favorable Healing, or Unfavorable Healing)	Teeth treated with minocycline exhibited no significant difference in the amount of favorable healing and the average remaining root structure when compared to those treated without minocycline
Chen et al. [21]	C: hemisected; extracted; filled with Gutta–Percha and sealer (AH plus); 60 min dry time; replanted; E: filled 3% Demeclocycline before 60 min dry time	3% Demeclocycline	Hematoxylin–eosin stain; the extent of root resorption and the type of interface between the root and surrounding tissue (Favorable Healing, or Unfavorable Healing)	the groups treated with Tetracycline had statistically significantly more favorable healing and more remaining root structure than the group filled with Gutta–Percha replanted after 60 min dry time
Gomes et al. [32]	C: extracted; dried for 60 min; removed the dental papilla; extirpated the pulp tissue through the apical foramen; removed the root surface-adhered PDL; filled the root canals with a calcium hydroxide–saline paste; sealed; replanted; E: additional systemic application of tetracycline	Tetracycline, 2.5 mg/kg, oral gavage, at 12-h intervals, for 7 days after tooth replantation	Hematoxylin–eosin stain; site of epithelial reattachment, PDL organization, intensity and extent of the acute and chronic inflammatory process at the site of epithelial reattachment and PDL, root resorption (active or inactive, extent, depth and repair), bone tissue, and ankylosis	Systemic antibiotic therapy has a positive influence on the repair process in delayed tooth replantation, but it was not statistically different
Melo et al. [33]	C: extracted; immersed in saline for 5 min and replanted E: additional systemic application of tetracycline	Intragastric administration of tetracycline, 2.5 mg/kg, 12/12 h, for 7 days after tooth replantation	Hematoxylin–eosin stain; site of epithelial reattachment, PDL organization, intensity and extent of the acute and chronic inflammatory process at the site of epithelial reattachment and PDL, root resorption (active or inactive, extent, depth and repair), bone tissue, and ankyloses	Systemic antibiotic therapy presented a positive effect in the immediate tooth replantation repair process, contributing to a better pulpal and PDL repair, but it was not statistically different
Bjorvatn et al. [36]	C: extracted; dry for 45 min and replanted E: immersed in doxycycline for 5 min, rinsed with saline before replantation	1% solution of doxycycline	Hematoxylin–eosin stain; the presence of uncomplicated healing, surface resorption, inflammatory resorption, replacement resorption (ankylosis), and inflammatory reaction in the absence of resorption	The application of doxycycline to the root surface had results similar with the control group
Selvig et al. [35]	C: extracted; root-planted; dry for 45 min and replanted E: immersed in tetracycline HCl for 5 min before replantation	1% solution of tetracycline HCl	Hematoxylin–eosin stain; the presence of surface resorption, inflammatory resorption, ankylosis, or inflammatory reaction in the absence of resorption	In control group, periodontal healing was characterized by massive ankylosis and inflammatory resorption. Experimental group showed great variation in healing response. Four teeth showed normal healing, whereas the remaining four teeth showed various amounts of inflammatory resorption, ankylosis, and persisting inflammation in the absence of resorption or ankylosis
Liu et al. [50]	C: immersed in saline for 5 min before replantation E: immersed in doxycycline for 5 min before replantation	0.05 mg/mL doxycycline	Clinical observation of the avulsed teeth	Compared with treatment with normal saline, avulsed permanent teeth treated with doxycycline did not show a better clinical outcome
Nam et al. [51]	C: extraction; dry for 5 min or preserved in HBSS for 60 min; saline irrigation for 10 s; replantation E: extraction; dry for 5 min or preserved in HBSS for 60 min; saline irrigation for 10 s; 5 min soaking in doxycycline before replantation; replantation	1 mg doxycycline in 20 ml saline	Micro-CT and hematoxylin–eosin stain; the presence of periapical radiolucency feature, the severity of root resorption	Pulpal healing in the doxycycline group was comparable with the control group. And the doxycycline group showed no improvement in periodontal healing after 5 min of dry storage, but exhibited a lower a lower grade of surface root resorption and inflammatory resorption in the teeth stored for 60 min in HBSS

C, control group; E, experimental group

### Pulpal healing

Five articles evaluated the effect of tetracyclines on pulp revascularization in replanted teeth [23, 24, 28, 29, 50].

#### Topical application

Yanpiset [29] and Cvek [24] revealed that topical application of doxycycline increased the frequency of complete pulp revascularization, while Ritter [28] showed the number of vital teeth in doxycycline-treated specimens was higher than saline-treated specimens, but no statistical significance was observed. Ritter [28] also illustrated that minocycline-treated specimens presented a significantly more number of vital tooth than saline-treated specimens. Liu [50] found avulsed permanent teeth treated with doxycycline did not show a better clinical outcome for pulp survival.

#### Systemic application

Cvek [23] illustrated that intravenous administration of doxycycline exerted no effect on the occurrence of complete pulp revascularization in reimplanted teeth.

### Periodontal healing

Twelve papers reported the effect of tetracyclines on periodontal healing in replanted teeth.

#### Topical application

The frequencies of ankylosis and inflammatory root resorption was statistically decreased in immature teeth soaked in doxycycline solution [24]. Replanted mature teeth filled with demeclocycline in the root canals showed significantly more favorable healing than the roots filled with Gutta–Percha [21]. The inhibitory effect of topical application of minocycline and tetracycline on root resorption was not significantly different from the control group [27, 34–36]. Nam [51] found that doxycycline improved the periodontal healing of replanted teeth stored for 60 min in HBSS, whereas doxycycline did not improve periodontal healing of replanted tooth after 5 min of dry storage. Only one human study showed that periodontal healing was similar between the teeth treated with normal saline and the one treated with doxycycline [50].

#### Systemic application

The inflammatory reaction was less intense and the complete healing was more common in the tetracycline group administered by oral gavage compared with control group, but the difference was also not statistically significant, no matter in the mature teeth [26] or in the rat incisors, with a wide apical foramen simulating the open apex of immature teeth [32, 33]. Mature teeth with dental plaque filled in the root canals showed significantly more complete healing and less inflammatory root resorption after oral administration of tetracycline than the control group [25].

## Discussion

Pulpal and periodontal healing are two main concerns of delayed replantation of avulsed teeth, and immediate replantation is recommended for the purpose of getting the success of pulp revascularization and periodontal ligament healing [4, 5].

### Root resorption and bacterial invasion

In avulsed teeth, mechanical trauma causing periodontal damage, might initiate the root resorption. This process could lead to removal of cementoblasts, precementum and sometimes cementum in areas of the root surface. Pulp necrosis, secondary to the displacement of teeth, makes necrotic pulp tissue much more vulnerable to microorganisms, which can reach the root canal through enamel-dentin cracks and exposed dentinal tubules. Bacteria and their irritants from the infected root canal could penetrate to the root surface through the dentinal tubule [52]. Damaged predentin and precementum might maintain and aggregate resorptive process [53]. Bacterial contamination left on the root surface and socket might also result in root resorption.

### Possible mechanisms of tetracyclines

The process of tooth resorption is considered to be similar to that of bone resorption. It is caused by osteoclasts, which are large and multinucleated cells, differentiated from hematopoietic mesenchymal stem cells along monocyte and macrophage lineage [54]. Antibiotics are recommended after tooth replantation in most situations and tetracyclines are the first choice [55]. Rifkin et al. [56] tried to illustrate the mechanism behind these activities and found that tetracyclines might inhibit bone resorption by decreasing osteoclast ruffled border, diminishing acid production and secreted cysteine proteinases, inducing cell retraction, elevating intracellular calcium, scavenging reactive oxygen species and possible inhibition of osteoclast collagenase. At the same time, they could exert influence on osteoblasts by increasing alkaline phosphatase and collagen synthesis to promote bone formation. Tetracyclines can also promote fibroblast and connective tissue attachment, enhancing regeneration of periodontal attachment [48].

The effect of tetracyclines is related to the concentration applied. In comparatively low concentration, known as subantimicrobial dose, they can inhibit the activity of matrix metalloproteinases and collagenase, and their degradation of non-osseous and osseous connective tissues [57]. Non-antibacterial tetracycline formulations have been used in periodontitis, dermatologic diseases, arthritis, cardiovascular disease [58–61]. In normal therapeutic concentrations, they have antibacterial activity by inhibiting bacterial protein synthesis and provide action against anaerobes, facultatives, rickettsia, mycoplasmas, chlamydia, and against a wide range of Gram-positive and Gram-negative organisms. However, a local application of high concentrations may cause tissue damage [62].

Tetracycline and its derivatives can strongly adsorb to tooth surfaces and then be slowly released in active forms, a property which prolongs therapeutic effectiveness [63]. In the research conducted by Cvek, pulp revascularization was significantly higher in group with the topical application of doxycycline than the control group [24]. This might be related to the antibacterial effect of doxycycline. Modified Gram stain was used to detect possible existence of micro-organisms in the pulpal lumen and dentinal tubules in this study. The results yielded that the frequency of micro-organisms was lower in the experimental group than control group. When the teeth with micro-organisms were excluded from the comparison, no difference of pulp revascularization was found between groups. This outcome indicated that the lower prevalence of bacterial in the pulpal lumen was responsible for the higher frequency of revascularization in the experimental group of teeth. Another result strengthened this point further. That is, fewer microorganisms were found in the group of immediately reimplanted teeth compared to the species with 30- and 60-min extra-alveolar time intervals, and a higher frequency of complete revascularization was demonstrated for the group of teeth reimplanted immediately [23, 24]. Another study showed that pulp revascularization occurred in 73% and 33% of the specimens in doxycycline-treated group and control group, separately. But the difference was not statistically significant. The small sample size might be responsible for this result. Whether there exist other reasons for this inconsistent outcome needs further study.

### Application of tetracyclines in tooth replantation

Cvek et al. reported that the topical application of doxycycline on tooth surface would facilitate the periodontal healing [24]. This might be related to the high frequency of complete pulp revascularization of these immature teeth ascribed to the use of doxycycline in the experimental group. Bacteria and their irritants from the infected root canal of necrotic pulp might participate in the resorptive process and hinder periodontal healing [52].

In the included studies of systemic application of tetracyclines, the dosages were 2 mg/kg in monkeys [23], 2.5 mg/kg in rats [32, 33] and 20 mg/kg in dogs [25, 26]. According to the equivalent dose translation between human and animal studies based on the body surface area (BSA) normalization method [64], the dosage for rats was subantimicrobial, while these for monkeys and dogs were antimicrobial. Four studies showed that systemic administration of tetracyclines could exert favorable effect on pulpal and periodontal healing in individuals, but the difference was not statistically significant between groups, no matter subantimicrobial or antimicrobial doses were used [18, 21, 27, 28]. And the results between mature and immature teeth were similar. The most common routes of infection after tooth replantation are the gingival tissues, periodontal tissues, main root canal, lateral canals, and dentinal tubules. Cvek et al. [23] demonstrated that the micro-organisms after tooth replantation were mainly seen in contaminated blood clots, located in the apical portion of the pulpal lumen or between abscesses below the vital tissue and the necrotized pulp. These bloods were formed during the injury and pushed into the pulpal lumen during the reimplantation. The amount of antibiotics diffusing into the replanted teeth after systemic administration is low, so it is difficult to play a role in inhibiting the growth and spread of the bacteria. Meanwhile, the variable absorption efficiency by oral gavage in different cases might contribute to the highly inconsistent results. However, A study carried out by Sae-Lim [25] showed that systemic tetracycline given orally could result in more complete periodontal healing and less inflammatory root resorption than blank control group. Root canals were filled with dental plaque and roots were shaved in this study, making it difficulty in comparing with others.

Filling the extracted roots with 3% Demeclocycline has been reported to be effective in facilitating more favorable healing and more remaining root structure than roots filled with Gutta–Percha [21]. But, possible side effect of this kind of root canal medicaments should be taken seriously since there is evidence suggesting that Ledermix (a paste containing triamcinolone and demeclocycline) might cause a darkening and gray-brown discoloration, which is unacceptable to patients [65, 66]. This adverse reaction is common during long-term use of the drugs [67]. Tsilingaridis et al. [30] applied doxycycline topically on avulsed permanent teeth in children, and there were no records pointing out that storing the avulsed teeth in a suspension of 1 mg doxycycline solution for 5 min will cause tooth discoloration. Systemic application of tetracyclines is often within one week, which is too short to produce any discoloration of the developing teeth [68]. Meanwhile, tooth replantation is advisable, since we should give priority to facial growth and development over esthetics [69].

### IADT guidelines

The International Association of Dental Traumatology (IADT) recommended systemic administration of antibiotics after avulsion and replantation to prevent infection-related reactions and to decrease the occurrence of inflammatory root resorption even though the value of systemic administration of antibiotic is highly questionable. Besides amoxicillin and penicillin, tetracycline and doxycycline are alternative antibiotics recommended after avulsion and replantation. And a specific topical antibiotic, duration of use, or methods of application is not recommended [55].

The effect of amoxicillin on healing of periodontal tissue was inconsistent compared to the treatment of tetracycline. Gomes [32] and Melo ME [33] found the effect of systemic amoxicillin on periodontal ligament repair was better than tetracycline, while studies conducted by Sae-Lim V [25, 26] illustrated the opposite result. The dosages of tetracycline used were 2.5 mg/kg in the former two studies and 20 mg/kg in the latter two studies, while the dosages of amoxicillin were 25 mg/kg and 22 mg/kg, respectively. This difference of applied dosages could affect the topical concentration of medicaments and the effect of antibiotics further, leading to contrary results.

The foremost limitation in this review is that only one human study was available [50]. Meanwhile, the administration, category, dosage of tetracyclines vary in different studies, which might reduce the comparability. This shortcoming confines the guidance of this systematic review. Hence, more studies are required to further estimate the effect of tetracyclines on pulpal and periodontal healing after tooth replantation.

## Conclusions

As a result of data heterogeneity and limitations of the studies, the effect of topical or systemic application of tetracyclines on pulpal and periodontal healing is inconclusive. More studies are required to get more clinically significant conclusions.

## Data Availability

The summary of data extraction in this study is available upon request to the corresponding author.

## Abbreviation

PRISMA: Preferred Reporting Items for Systematic Reviews and Meta-Analyses
